# Growth of GeSi nanoislands on nanotip-patterned Si (100) substrates with a stress-induced self-limiting interdiffusion

**DOI:** 10.1186/1556-276X-7-346

**Published:** 2012-06-26

**Authors:** Ruifan Tang, Kai Huang, Hongkai Lai, Cheng Li, Zhiming Wu, Junyong Kang

**Affiliations:** 1 Department of Physics, Xiamen University, Xiamen, 361005, China

**Keywords:** GeSi nanoislands, Nanotip pre-patterned Si substrates, Ge concentration distribution, Stress-induced interdiffusion self-limiting effect, PACS, 68.35.Gy, 61.46.-w, 66.30.Pa

## Abstract

GeSi nanoislands grown on nanotip pre-patterned Si substrates at various temperatures are investigated. Nanoislands with a high density and narrow size distribution can be obtained within an intermediate temperature range, and the Ge atom diffusion length is comparable to half of the average distance of the Si nanotips. The Ge concentration distributions at the center and edge of the GeSi nanoislands are measured by scanning transmission electron microscopy. The results reveal that there is a Si core at the center of the GeSi nanoisland, but the Ge concentration presents a layered distribution above the Si nanotips. The radial component of the stress field in Ge layer near the Ge/Si interface on the planar, and the nanotip regions is qualitatively discussed. The difference of the stress field reveals that the experimentally observed concentration profile can be ascribed to the stress-induced interdiffusion self-limiting effect of the Si nanotips.

## Background

The self-organized growth of semiconductor nanodots has attracted considerable interest. Fundamental investigations on their structural, electronic, and optical properties, as well as on their potential use in electronic and optoelectronic devices, have been extensively conducted [1-5]. Among the broad range of semiconductor material systems, group IV semiconductor (Si and Ge) nano/heterostructures are distinctive in that they offer material compatibility and facilitate integration with well-established Si-based technology [6-13]. Ge possesses a number of properties superior to those of Si in device applications, e.g., higher carrier mobility and larger exciton radius, which result in a stronger quantum confinement within the nanostructure and the possibility of low processing temperatures. Thus, Ge can be easily integrated with conventional devices. Among the fabrication methods of Ge nanostructures, the strain-driven formation of Ge nanoislands on Si during epitaxial growth has been wide studied for both plain and patterned substrates. This process is a promising way of fabricating self-assembled nanoislands via the Stranski–Krastanov growth mode [2,14-18]. On plain substrates, islands nucleate on randomly distributed sites. The morphological island evolution depends on a subtle interplay between kinetics and thermodynamics set by the growth conditions [19]. The random character of the formation process results in a broad distribution of island size and concentration [20].

To narrow down the statistical size distribution of Ge nanoislands, several methods of predefining the nucleation sites have been reported, including buried stressors [21,22], pre-patterned SiO_2_ layers [6], as well as pit- [8,12] and stripe-patterned substrates [23]. However, for most of the pre-patterned methods, a sub-100 nm distance of nucleation sites is difficult to reach. Thus, the growth temperature of Ge nanoislands with a narrow size distribution must be sufficiently high to match the diffusion length of Ge ad-atoms on Si and the distance of nucleation sites.

In the present study, nanotip pre-patterned Si substrates with an average intertip distance of 50 nm are fabricated by anodic aluminum oxide (AAO) assistant etching and subsequent thermal desorption in an ultra-high vacuum chemical vapor deposition (UHVCVD) system. Using these nanotip pre-patterned Si substrates, GeSi nanoislands with a high density and narrow size distribution are obtained when the growth temperature is 500°C. The different Ge concentration distributions at the center and edge of the GeSi nanoislands are also investigated. The results reveal that there is a Si core at the center of the GeSi nanoisland, but the Ge concentration presents a layered distribution above the Si nanotips. The mechanism for determining the Ge concentration profile on the Si nanotips is discussed.

## Methods

The samples were prepared from n-type (100) Si wafers with 0.1 to 1.2 Ωcm resistivity. The fabrication methods of the Si-based nanotips on Si substrates were the same as those detailed in our previous publication [24]. To reduce further the size of the nanotips on Si substrates, the samples were treated with a standard Radio Corporation of America (RCA) cleaning process and thermal desorption procedure at 850°C in an ultra-high vacuum. The nanotip pre-patterned Si substrates were then formed. The surface morphology of AAO was observed by a scanning electron microscopy (SEM) system (LEO 1530, Carl Zeiss AG, Oberkochen, Germany). The surface morphologies of Si-based nanotips on Si substrates and the nanotip pre-patterned Si substrates were examined by an atomic force microscopy (AFM) system (Nanoscope IIIa, Veeco Instruments Inc., Plainview, NY, USA**.**

The GeSi nanoislands were grown by UHVCVD epitaxy using the nanotip pre-patterned Si substrates described above directly, i.e., there is not Si buffer layer deposited prior to the GeSi nanoislands growth. GeSi islands were grown using pure germane (GeH_4_) as the gas source with a flow rate of 1 sccm at 450, 500, and 550 °C for 4 min for samples A, B, and C, respectively. The growth rates of samples A, B, and C are approximately 5.5, 6.3 and 7.0 ML/min, respectively. The surface morphologies of the GeSi nanoislands were investigated by AFM. For sample B, the cross-sectional structure of the GeSi islands and concentration distribution at the center and edge position of the GeSi islands were investigated by transmission electron microscopy (TEM) and scanning transmission electron microscopy (STEM).

## Results and discussion

**Figure 1:**
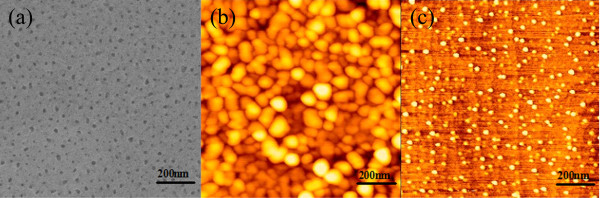
**SEM images and AFM images.** (**a**) SEM image showing the surface morphology of Si-based AAO, (**b**) AFM image of Si substrates after eroding the AAO structure and standard RCA cleaning, (**c**) AFM image of the nanotip-patterned Si substrates.

The SEM image in Figure 1a presents the surface morphology of the AAO film on silicon substrate, and nanoscale hole arrays were observed. The density of the holes was approximately 3.8 × 10^10^ cm^−2^, the average diameter of the holes was approximately 20 nm, and the average distance between holes was approximately 50 nm. The AFM image in Figure 1b shows that the Si-based nanotip arrays were fabricated by the pattern transfer of the hole shape of anodic porous alumina after eroding the AAO structure and silicon oxide below the pores. The density of the tips was approximately 2 × 10^10^ cm^−2^, and the average size of the islands was 50 ± 10 nm. Afterwards, the samples were treated by a standard RCA process. AFM morphology (not shown) demonstrated that the morphology of the sample surface did not change after standard RCA processes. Considering that the RCA process creates a reproducible layer of native oxide, the nanotips can be reasonably identified as nanotips capped with a SiO_2_ layer. Figure 1c presents the AFM image of a Si nanotip on silicon substrate when the oxide layer was desorbed at 850°C in an ultra-high vacuum. Si nanotips with a large area and uniform distribution can be observed on the surface. The average nanotip size is 26 ± 8 nm, which is approximately half that of the Si-based nanotips shown in Figure 1b. The average height of the Si nanotips was approximately 1.3 ± 0.1 nm. The density of the Si nanotips is approximately 3.8 × 10^10^ cm^−2^, which is larger than that of the Si-based nanotips before RCA process. A thermal annealing process in an ultra-high vacuum can be described by the equation SiO_2_ + Si → 2SiO (gas). Hence, the SiO_2_ layer reacted with the Si atoms under the SiO_2_ layer to form gas phase SiO, which was then released [25,26]. Thus, the nanotip size decreased and formed Si nanotips. The possible reason for the increased density was the division of the large SiO_2_ islands during the reaction process. It can be measured from Figure 1c that the average distance between the Si nanotips was approximately 47 ± 8 nm, which was at the same order of magnitude as the surface diffusion length of Ge ad-atoms moving on patterned Si substrates. Thus, these nanotip pre-patterned Si substrates were used as the patterned substrates for the growth of self-assembling GeSi islands by the UHVCVD system.

Analogous to growth on planar substrates, the surface diffusion length *L* of ad-atoms moving on patterned substrates is given by L=(Dτ)12,D=a2vexp−EKBT[8]. *D* is the diffusion constant, *τ* is the time interval for ad-atom diffusion, *a* is the lateral motion (3.84 A) corresponding to each hop of an ad-atom diffusion, *v* is a prefactor (about 10^13^), *E* is the effective barrier for ad-atom diffusion on patterned substrates, *K*_*B*_ is the Boltzmann constant, and *T* is the growth temperature. *E* was assumed to be 1.5 eV, and *τ* was adopted to be one quarter of the growth time of 1 ML (when, on the average, no nearest neighboring site is occupied by other atoms around each ad-atom to avoid the interaction between ad-atoms during diffusion). The estimated diffusion lengths (*L*) can be calculated as 11, 23 and 45 nm at the growth temperatures of 450, 500 and 550°C, respectively.

**Figure 2:**
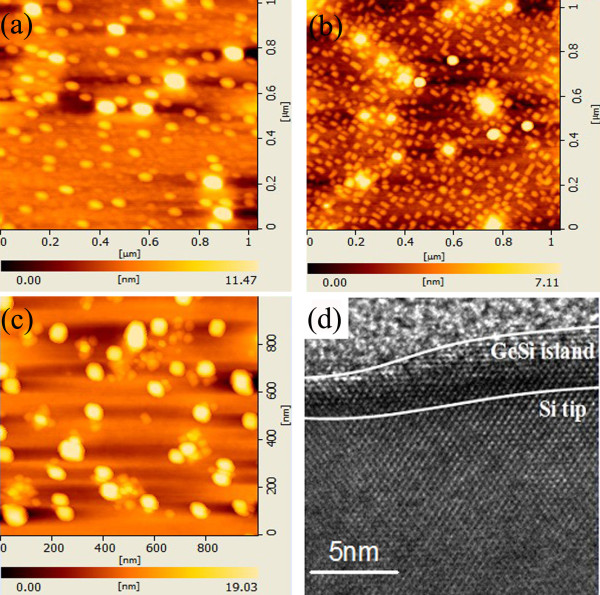
**The surface morphology of GeSi islands.** Surface morphology of GeSi islands grown on the nanotip pre-patterned Si (100) substrates grown at (**a**) 450 °C for 4 min, (**b**) 500 °C for 4 min, and (**c**) 550 °C for 4 min; (**d**) the cross-sectional high-resolution TEM images of GeSi islands grown at 500 °C.

The AFM image in Figure 2a presents the surface morphology of sample A grown at 450°C on the nanotip pre-patterned Si substrates. The average diameter of the GeSi islands is approximately 60 ± 15 nm, and the density is approximately 1 × 10^10^ cm^−2^. GeSi islands are mostly dome-shaped and super dome-shaped, which is similar to the dual-mode distribution of Ge quantum dots on flat substrates [20]. This result can be attributed to the relatively low growth temperature. When the growth temperature is as low as 450°C, the diffusion length *L* (11 nm) is much smaller than half of the average distance *d/2* (23.5 nm) between Si nanotips. Thus, most of the deposited Ge ad-atoms cannot diffuse onto the pre-patterned nanotips on Si substrates. Accordingly, the inducement action of the nanotip pre-patterned substrates on the growth of GeSi islands did not present obviously. GeSi islands grow all over the surface and exhibit a broad size distribution similar to those on planar substrates.

The AFM image in Figure 2b illustrates the surface morphology of sample B grown at 500°C, and GeSi islands with uniform size and distribution can be observed. The GeSi islands are rectangular with widths of approximately 30 ± 2 nm and a density of approximately 5 × 10^10^ cm^−2^, which was larger than that of the Si nanotips shown in Figure 1c. This finding may be related to the influence of the Si nanotips. At an intermediate temperature of 500°C, *L* can be calculated to be 23 nm, which was comparable to *d/2*. Most deposited Ge ad-atoms can diffuse into the corresponding nanotips on the Si substrate [27], where the strain energy is relatively low, and then form GeSi islands [28]. This mechanism was also supported by the cross-sectional high-resolution TEM image of sample B in Figure 2d. A GeSi island can be observed at the top of the Si nanotip. The height of the GeSi islands is approximately 5 nm, which well agrees with the AFM image. Given that *d* is the average distance between Si nanotips, the distance between part of the Si nanotips can be assumed to be larger than *d*. Thus, part of the Ge ad-atoms cannot diffuse onto the nanotips, and they form GeSi islands on the planar region of the Si substrate. Consequently, the density of the GeSi islands was larger than that of the Si nanotips. The morphology of the GeSi islands is very similar to the hut clusters described in previous reports [29]. According to the reference, we believe that the island edges are oriented along <100 > directions.

Figure 2c shows the AFM image of sample C, which was grown at the relatively high temperature of 550°C. The average diameter increased to approximately 70 ± 20 nm, and the density decreased to approximately 0.5 × 10^10^ cm^−2^. These results can be attributed to the larger *L* (45 nm) at the relatively high growth temperature of 550°C, which is very close to *d*. In the previous studies, ordered islands will grow when *L* is comparable to *d*[8]. We think that the difference of our experiment and the previous studies might be due to the pre-pattern methods. In our case, the height of the nanotips is as small as 1.3 nm, thus the difference of the strain energy on the planar and the nanotip regions is not sufficient for influencing the nucleation of the GeSi nanoislands obviously at a relatively high temperature.

**Figure 3:**
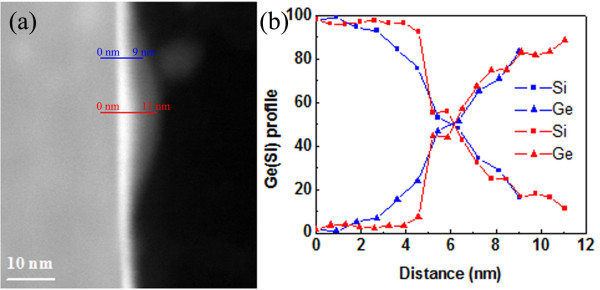
**The STEM image of the distribution and the EDX line scan.** (**a**) STEM image of the distribution of components at the center and edge positions of GeSi islands, (**b**) EDX line scan taken along the indicated lines shown in the STEM image. EDX data were acquired along the lines near the center (red line) and edge (blue line) of the islands.

To evaluate the composition distribution of GeSi islands grown on nanotip pre-patterned Si substrates, the STEM image and EDX line scan taken along the indicated lines are shown in Figure 3a,b, respectively. EDX data were acquired along the lines near the center (red line) and edge (blue line) of the islands (Figure 3a). For the EDX data obtained from the edge of the island, the Ge concentration began to increase at about 2 nm, which can be defined as the nominal GeSi/Si interface. The Ge concentration increased rapidly near the nominal interface at the measuring region between 2 and 5 nm with a slope of approximately 13% per nanometer. The slope of the Ge concentration profile then decreased to approximately 9% per nanometer. This can be attributed to the Ge/Si interdiffusion. The two-stage increase in Ge concentration can be explained by the more difficult diffusion of Ge atoms into the Si substrate than that of Si atoms into the Ge layer. However, for the EDX data obtained from the center of the GeSi islands, the Ge concentration began to increase at about 4 nm, which indicated a 2 nm-high Si core without any Ge at the center of the GeSi island. The Ge concentration increased from 4 to 5 nm with a slope of approximately 43% per nanometer, which was much larger than the slope of the profile from the edge of the GeSi island. The slope of the Ge concentration then decreased to approximately 9% per nanometer at the measuring region between 5 and 9 nm, similar with the slope of the profile from the edge of the GeSi island. At the measuring region between 9 and 11 nm, the slope of the Ge composition profile further decreased to 3% per nanometer. These data indicated that at the center of the GeSi island, the Ge concentration increased rapidly to approximately 45% within 1 nm just above the Si core. Thereafter, the Ge concentration increased as slowly as that at the edge of the GeSi island. At the same measuring distance, the Ge concentration at the edge of the GeSi island was larger than the Ge concentration at the center of the GeSi island when the distance was between 2 and 5 nm. However, the Ge concentrations at the edge and center of the GeSi islands were almost the same when the measuring distance was between 5 and 9 nm. This non-uniform Ge concentration distribution can be attributed to the stress-induced Ge/Si interdiffusion self-limiting effect on the Si nanotip pre-patterned Si substrates.

**Figure 4:**
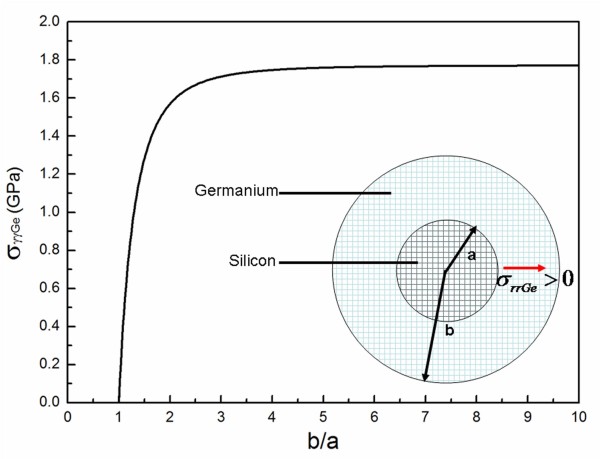
**The radical component of the stress field vs. the ration of the Ge shell radius.** The radial component of the stress field in Ge shell near the Ge/Si interface vs. the ratio of the Ge shell radius to the Si core radius (b/a). Insert shows a schematic diagram of the system.

The radius of cuvature of the planar and nanotip region is different. When Ge layer was deposited on the substrate, for simplicity, the structure of Ge/Si is regarded as a Si core epitaxially capped with a concentric Ge shell. A schematic diagram of the system is shown in insert of Figure 4. The inner region, 0<r<a, is the Si core and the outer region, a<r<b is the Ge shell. For Ge layer deposited on a planar region, a=b=∞. For Ge layer deposited on a nanotip region, a<b<∞. The diffusion of the Ge atoms into the Si substrate is driven by the radial component of the stress field and the concentration gradient. According to the reference [30], the radial component of the stress field in the Ge shell vary according to the distance from the center of the Si core,$\sigma_{\text{rr}G\text{e}}\text{=}\frac{\text{cp}}{\left(1-c\right)}\left[1-{\left(\frac{\text{b}}{\text{r}}\right)}^{3}\right]$ Where $\text{c =}\frac{{\text{a}}^{3}}{{\text{b}}^{3}}$ is the volume fraction of the Si core, $\text{p}=\frac{\frac{2\epsilon_{\mathrm{Ge}}}{3(1-\upsilon_{\mathrm{Ge}})}\epsilon (1-c)}{1-\frac{2m}{3}(1-c)}$ is the hydrostatic stress inside the Si core, i.e., the interface press [31]$m=\frac{\epsilon_{\mathrm{Ge}}}{1-\upsilon_{\mathrm{Ge}}}\left[\frac{\left(1-2\upsilon_{\mathrm{Ge}}\right)}{\epsilon_{\mathrm{Ge}}}-\frac{\left(1-\upsilon_{\mathrm{Si}}\right)}{\epsilon_{\mathrm{Si}}}\right]$ is the elastic mismatch parameter and$\epsilon =(\frac{3\kappa_{\mathrm{Si}}}{3\kappa_{\mathrm{Si}}+4\mu_{\mathrm{Ge}}})(\frac{\alpha_{\mathrm{Si}}-\alpha_{\mathrm{Ge}}}{\alpha_{\mathrm{Ge}}})$ is the constrained strain for a spherical geometry [32,33]. The constrain strain is calculated assuming that the lattice parameter of Si core is the same as in bulk.$\epsilon_{G\text{e}}\text{=}1.02\times 10^{11}$ Pa, $\epsilon_{S\text{i}}\text{=}1.31\times 10^{11}$ Pa is the Young's modulus of Ge and Si, respectively, $\upsilon_{G\text{e}}\text{=}0.278$$\upsilon_{S\text{i}}\text{=}0.29$ is the Poison's ratio of Ge and Si, respectively, $\kappa_{S\text{i}}\text{=}0.96\times 10^{11}$ Pa is the Bulk modulus of Si and $\mu_{G\text{e}}\text{=}0.67\times 10^{11}$ Pa is the shear modulus of Ge. Thus, the radial component of the stress field in Ge shell near the Ge/Si interface can be calculated as:

(1) $$\sigma_{\mathrm{rrGe}}=-\frac{1.95\times 10^{9}c}{1+0.1\times (1-c)}\left[1-{\left(\frac{b}{a}\right)}^{3}\right]$$

$\sigma_{\mathrm{rrGe}}$ is positive when b>a. The positive stress indicates that the stress will prevent Ge atoms from diffusing into Si core. It can be deduced that the radial component of the stress field in Ge shell near the Ge/Si interface is a function of the ratio of the shell radius to the core radius (γ≡ba) (Figure 4). $\sigma_{\text{rr}Ge}=0$ when γ=1 is the radial component of the stress field in Ge layer epitaxied on a planar Si substrate. Considering the driven force of the concentration gradient, the interdiffusion of Ge and Si occurs easily. But when γ>1$\sigma_{\mathrm{rrGe}}$ increases rapidly. While the driven force of the concentration gradient does not change with the geometry, the interdiffusion of Ge and Si will be self-limited on Si nanotip region.

**Figure 5:**
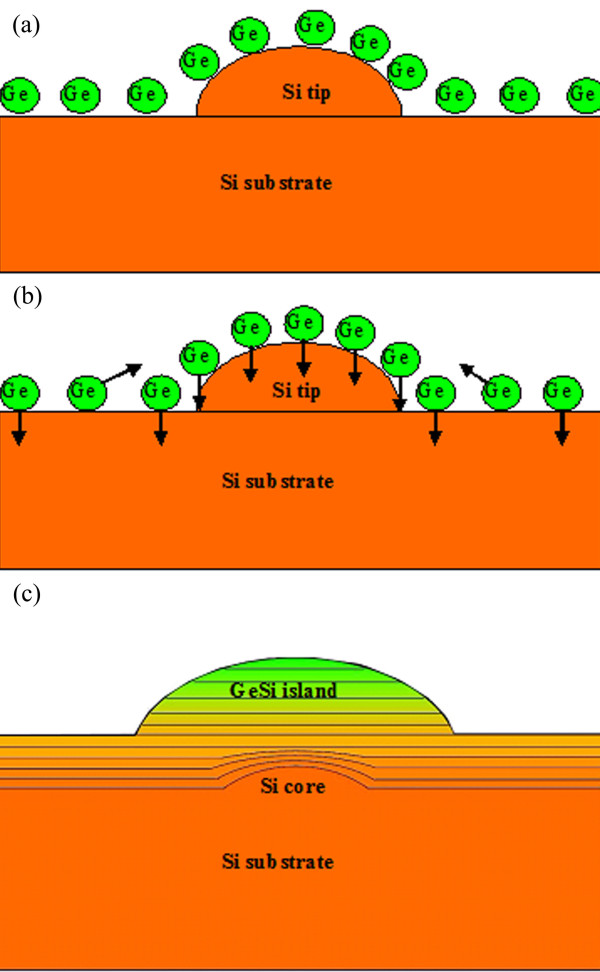
**Growth model of Ge islands on the nanotip pre-patterned Si substrate:** (**a**) Ge atoms were deposited onto the Si nanotips and planar region, (**b**) some Ge atoms diffuse laterally to the corresponding nanotips on Si substrate, where the strain energy is relatively low, and then form GeSi islands. At the same time, other Ge atoms diffuse vertically into the Si substrates, (**c**) the composition gradient and layer structure of GeSi islands formed on the Si core.

The possible schematic images of GeSi nanoislands grown on Si nanotip pre-patterned substrates are shown in Figure 5. First, a layer of Ge atoms is deposited on both the planar and nanotip regions, as shown in Figure 5a. After the deposition of Ge atoms, two processes occur simultaneously (Figure 5b). The Ge atoms diffuse laterally to the corresponding nanotips on the Si substrates, where the strain energy is relatively low, and then form GeSi islands. At the same time, the Ge atoms diffuse vertically into the Si substrates. As discussed above, on the planar region, $\sigma_{\text{rr}Ge}=0$. Thus, the concentration gradient of Ge was uniform through the whole interdiffusion layer. But on the region near the Si nanotip, $\sigma_{\text{rr}Ge}>0$. Thus, the concentration gradient of Ge was much bigger than that on the planar region (Figure 5c).

The EDX data also indicated that the Ge concentrations at the center and edge of the GeSi nanoislands were almost the same at the region between 5 and 9 nm. Accordingly, the Ge concentration presented a layered distribution above the Si nanotips. This finding can be explained by the lateral diffusion of Si atoms into the GeSi layer. For the Si nanotip region, the stress-induced interdiffusion self-limiting effect prevented the Ge atoms from diffusing into the Si nanotips. At the same time, the Si atoms were prevented from diffusing into the GeSi layer. Thus, the amount of Si atoms diffusing from the Si nanotips into the GeSi layer were much lower than the amount of Si atoms diffusing from the planar Si substrate into the GeSi layer. There may be a Si concentration gradient in the GeSi layer between the planar Si and nanotip Si regions. Accordingly, the lateral diffusion of Si atoms occurred above the Si nanotips, and the Ge concentrations were uniform on the planar Si and nanotip Si regions.

## Conclusions

Uniform self-assembling GeSi nanoislands were fabricated using nanotip pre-patterned Si substrates. The unique features of the GeSi nanoislands were investigated as a function of the growth temperature. These findings were discussed in terms of the surface diffusion length *L* compared with half of the average distance of Si nanotips on the substrate. The different Ge concentration distributions at the center and edge of the GeSi nanoislands were also investigated by EDX spectra. The results revealed a Si core at the center of the GeSi nanoisland, but the Ge concentration presented a layered distribution above the Si nanotips. The discussion of the radial component of the stress field in Ge layer near the Ge/Si interface on the planar and the nanotip regions can qualitatively explain the experimentally observed Ge concentration distributions at different regions.

## Abbreviations

AAO: anodic aluminum oxide; UHVCVD: ultra-high vacuum chemical vapor deposition; RCA: Radio Corporation of America; SEM: scanning electron microscopy; AFM: atomic force microscopy; TEM: transmission electron microscopy; STEM: scanning transmission electron microscopy; L: diffusion lengths.

## Competing interests

The authors declare that they have no competing interests.

## Authors' contributions

RT carried out the experiments studied on the GeSi nanoislands growth and drifted the manuscript. Dr. KH designed the research programs and guided the experiment's progress. Professors HL, CL, ZW, and JK participated in the mechanism development. All authors read and approved the final manuscript.

## Authors' information

RT is a postgraduate student on physics. He researches on the growth of GeSi materials at Xiamen University. Dr. KH works as an associate professor at the Xiamen University. He works on fabrication and the optics behaviors of nanomaterials. Professors HL, CL, ZW, and JK are experts of physics at Xiamen University.
